# Early release of high mobility group box nuclear protein 1 after severe trauma in humans: role of injury severity and tissue hypoperfusion

**DOI:** 10.1186/cc8152

**Published:** 2009-11-04

**Authors:** Mitchell J Cohen, Karim Brohi, Carolyn S Calfee, Pamela Rahn, Brian B Chesebro, Sarah C Christiaans, Michel Carles, Marybeth Howard, Jean-François Pittet

**Affiliations:** 1 The Department of Surgery, San Francisco General Hospital, University of California San Francisco, 1001 Potrero Avenue, San Francisco, CA 94110, USA; 2 The Royal London Hospital, Whitechapel, London E1 1BB, UK; 3 The Department of Medicine, San Francisco General Hospital, University of California San Francisco, 1001 Potrero Avenue, San Francisco, CA 94114, USA; 4 The Department of Anesthesia, San Francisco General Hospital, University of California San Francisco, 1001 Potrero Avenue, San Francisco, CA 94110, USA

## Abstract

**Introduction:**

High mobility group box nuclear protein 1 (HMGB1) is a DNA nuclear binding protein that has recently been shown to be an early trigger of sterile inflammation in animal models of trauma-hemorrhage via the activation of the Toll-like-receptor 4 (TLR4) and the receptor for the advanced glycation endproducts (RAGE). However, whether HMGB1 is released early after trauma hemorrhage in humans and is associated with the development of an inflammatory response and coagulopathy is not known and therefore constitutes the aim of the present study.

**Methods:**

One hundred sixty eight patients were studied as part of a prospective cohort study of severe trauma patients admitted to a single Level 1 Trauma center. Blood was drawn within 10 minutes of arrival to the emergency room before the administration of any fluid resuscitation. HMGB1, tumor necrosis factor (TNF)-α, interleukin (IL)-6, von Willebrand Factor (vWF), angiopoietin-2 (Ang-2), Prothrombin time (PT), prothrombin fragments 1+2 (PF1+2), soluble thrombomodulin (sTM), protein C (PC), plasminogen activator inhibitor-1 (PAI-1), tissue plasminogen activator (tPA) and D-Dimers were measured using standard techniques. Base deficit was used as a measure of tissue hypoperfusion. Measurements were compared to outcome measures obtained from the electronic medical record and trauma registry.

**Results:**

Plasma levels of HMGB1 were increased within 30 minutes after severe trauma in humans and correlated with the severity of injury, tissue hypoperfusion, early posttraumatic coagulopathy and hyperfibrinolysis as well with a systemic inflammatory response and activation of complement. Non-survivors had significantly higher plasma levels of HMGB1 than survivors. Finally, patients who later developed organ injury, (acute lung injury and acute renal failure) had also significantly higher plasma levels of HMGB1 early after trauma.

**Conclusions:**

The results of this study demonstrate for the first time that HMGB1 is released into the bloodstream early after severe trauma in humans. The release of HMGB1 requires severe injury and tissue hypoperfusion, and is associated with posttraumatic coagulation abnormalities, activation of complement and severe systemic inflammatory response.

## Introduction

Trauma remains the leading cause of mortality for patients between 1 and 40 years of age and eclipses cancer, heart disease and HIV/AIDS [1]. Although there remain a large proportion of trauma victims who die early from overwhelming injury, trauma patients who survive their initial injury do not die from their injury *per se*, but from an overwhelming inflammatory dysregulation leading to organ dysfunction, nosocomial infection, and ultimately multiorgan failure [2,3]. The mechanisms that initiate this sterile inflammatory process are still not completely understood.

It has been known for several years that severe trauma is associated with an early systemic inflammatory response syndrome (SIRS) followed by a compensatory anti-inflammatory response syndrome (CARS), although the molecular mechanisms responsible for this altered host defense are not well understood [3-5]. However, recent studies have provided new information on the molecular mechanisms that lead to this early inflammatory response. Complement and alarmins have been shown to play an important role as endogenous triggers of trauma-associated inflammation. The complement system appears to represent one of the key mediators of the innate immune response after ischemia-reperfusion and trauma [6-8]. Once activated through the Mannose Binding Lechtin pathway, the activation of complement is amplified via the alternative pathway [9,10]. Complement plays a critical role as a chemoattractant for phagocytes and polymorphonuclear leukocytes and recruits these immune cells to the site of injury. C3a and C5a bind to their receptors on endothelial cells eliciting an inflammatory response via the activation of the Mitogen-activated protein kinases. Finally, the generation of C5b by cleavage of C5 generates the membrane attack complex that can lyse eukaryotic cells [8,11].

The second class of early proinflammatory mediators is called alarmins and represents the correlate of pathogen-associated molecular patterns (PAMPs) for all non-pathogen-derived danger signals that originate from tissue injury [12,13]. These include heat shock proteins, annexins, defensins, S100 protein and high mobility group box nuclear protein 1 (HMGB1). These alarmins are endogenous molecules capable of activating innate immune responses as a signal of tissue damage and cell injury. Among the alarmins, HMGB1 is a DNA nuclear binding protein that has recently been shown to be involved in the triggering of sterile inflammation [14]. HMGB1 release has been described in both necrotic and apoptotic cells as well as via a non-classical pathway in immune and non-immune cells [14]. HMGB1 has become the archytypal mediator of cellular alarm after sterile stress or injury. For example, HMGB1 has been shown to both stimulate macrophages and endothelial cells to release TNF-α, IL-1 and IL-6 via the activation of several receptors including toll-like-receptor 4 (TLR4) and receptor for the advanced glycation end products (RAGE) [14-16].

Although the extracellular release of HMGB1 has been reported by several investigators in patients with infection and sepsis, only one study has described HMGB1 release in plasma in a small group of patients several hours after trauma [17]. It has been shown that HMGB1 is released early in the plasma of animals that undergo hemorrhagic shock and trauma and functions as one of the key mediators of the sterile inflammation induced by ischemia-reperfusion injury [18]. However, it is not known whether HMGB1 is also released in the plasma early after trauma in humans and this open experimental question constitutes the first aim of this study. Furthermore, because HMGB1 has been shown to induce microvascular thrombosis and endothelial cell activation [19] and because we have previously described an activation of the protein C and of the complement pathways that occurs nearly immediately after trauma [9,20], we also sought to define the relations between plasma levels of HMGB1, activation of coagulation and of the protein C system and the release of other markers of inflammation and endothelial activation early after trauma. Here, we report an extracellular release of HMGB1 within 30 minutes after trauma that correlates with severity of injury, tissue hypoperfusion, activation of the protein C system and coagulation abnormalities, complement activation and the release of other biomarkers of endothelial cell activation after severe trauma in humans.

## Materials and methods

The Institutional Review Board of the University of California at San Francisco approved the research protocol for this prospective cohort study and granted a waiver of consent for the blood sampling as it was a minimal risk intervention.

### Patients

Consecutive major trauma patients admitted to the San Francisco General Hospital (level one trauma center) were studied. All adult trauma patients who met criteria for full trauma team activation were eligible for enrollment. Patients less than 18 years old or transferred from other hospitals were excluded. In addition, patients with previous coagulation abnormalities were also excluded from the study.

### Sample collection and measurements

The methodology has been described previously in detail [20]. Briefly, a 10 ml sample of blood was drawn in citrated tubes within 10 minutes of arrival in the emergency department. The samples were immediately transferred to the central laboratory, centrifuged and the plasma extracted and stored at -80°C. Samples were analyzed at the conclusion of the study by researchers who were blinded to all patients data. In this study, we measured HMGB1 (HMGB-1 ELISA kit IBL, Transatlantic LLC, Osceola, WI, USA). These results were compared with IL-6, TNF-α (both from R&D Systems Inc., Minneapolis, MN, USA), von Willebrand Factor (vWF) antigen (Asserachrom vWF, Diagnostica Stago Inc., Parsippany, NJ, USA), angiopoietin-2 (Ang-2; Quantikine Ang-2 EIA, R&D Systems Inc., Minneapolis, MN, USA), soluble C5b-9 to assess the late phase of terminal complement activation (sC5b-9 EIA, Quidel Corp., San Diego, CA, USA), prothrombin fragments (PF 1+2; Enzygnost F1+2 EIA, Dade Behring, Germany), soluble thrombomodulin (TM; Asserachrom Thrombomodulin EIA, Diagnostica Stago Inc., Parsippany, NJ, USA), and plasminogen activator inhibitor 1 (PAI-1; Oxford Biochemicals, Oxford, MI, US). protein C activity, tissue plasminogen activator (t-PA), and D-Dimers were measured with a Stago Compact (Diagnostica Stago Inc., Parsippany, NJ, USA), All measurements were performed in accordance with the manufacturers' instructions.

### Data collection, outcome measures

Data were collected prospectively on patient demographics, the injury time, mechanism (blunt or penetrating) and severity, pre-hospital fluid administration, time of arrival in the trauma room and admission vital signs. The Injury Severity Score (ISS) was used as a measure of the degree of tissue injury [21]. An arterial blood gas was drawn at the same time as the research sample as part of the standard management of major trauma patients. The base deficit was used as a measure of the degree of tissue hypoperfusion. Admission base deficit is a clinically useful early marker of tissue hypoperfusion in trauma patients and an admission base deficit greater than 6 mmol/l has previously been identified as being predictive of worse outcome in trauma patients [22,23].

#### Outcome measures

Patients were followed until hospital discharge or death. For mortality analysis, patients surviving to hospital discharge were assumed to still be alive. Secondary outcome measures were also recorded for 28-day ventilator-free days, acute lung injury (American-European consensus conference definition) [24] and acute renal injury (Acute Dialysis Quality Initiative consensus conference definition) [25] and blood transfusions required in the first 24 hours.

### Statistical analysis

Data analysis was performed by the investigators. Normal-quantile plots were used to test for normal distribution. Relations between quartile of HMGB1 and continuous variables were tested with the Kruskall-Wallis test followed by a non-parametric test for trend. Two-group analysis was performed using the Wilcoxon rank-sum method. Correlation was assessed by Spearman correlation coefficients. Logistic regression was used to examine the relationship between mortality and HMGB1 levels. A *P *≤ 0.05 was chosen to represent statistical significance.

## Results

### Patient population

Table 1 shows the characteristics of the severely injured trauma patients enrolled in the study. We enrolled 168 consecutive traumatized patients into the study over a 15-month period. Due to short transport times from the scene of injury to our trauma center in San Francisco, the mean (± standard deviation) time from injury to blood sampling was 32 ± 6 minutes. Patients received an average of 150 ± 100 ml of intravenous crystalloid prior to blood sample collection, but did not receive any vasopressor, colloid or emergency blood prior to blood sample collection.

**Table 1:** Clinical characteristics of trauma patients

**Demographic data**	
Age, years	41(27-63)
Sex, female/male	n = 50 (30%)/n = 118 (70%)
**Characteristics on injury**	
Injury Severity Score	17 (9-26)
Penetrating injury	n = 43 (25%)
Severe head injury (AIS head >3)	n = 47 (27%)
**Physiology**	
Heart rate >100 beats/min	n = 76 (45%)
Systolic blood pressure <100 mmHg	n = 38 (22%)
Base deficit >6 mmol/l	n = 56 (27%)
**Blood samples**	
Time from injury to emergency department arrival (min)	28 (23-29)
Time from emergency department arrival to sample (min)	4 (1-9)
Intravenous fluids prior to initial blood sample (ml)	100 (0-200)

Total number of patients included is 168. Data are presented as median (interquartile range) and numbers (%). Time of injury is defined as the time of pre-hospital emergency medical service activation.

AIS: Abbreviated Injury Scale.

### Plasma levels of HMBG1 correlate with arterial base deficit and ISS score in trauma patients

**Figure 1:**
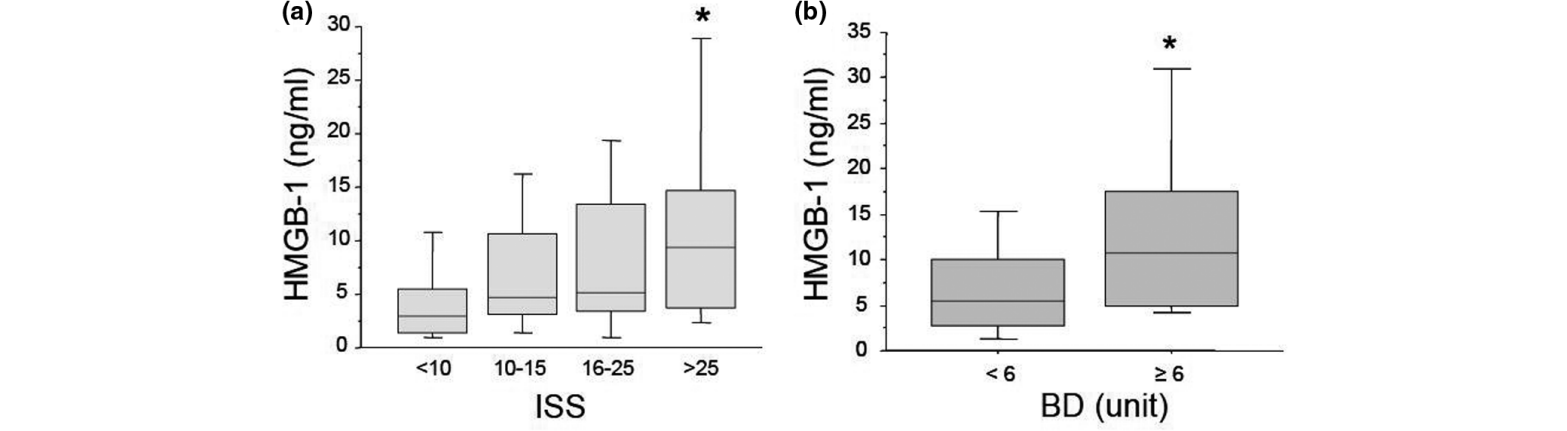
Effects of injury and arterial base deficit on plasma levels of HMGB1 early after trauma. Blood samples were obtained from 168 consecutive major trauma patients immediately upon admission to the hospital. **(a and b) **Plasma levels of high mobility group box nuclear protein 1 (HMGB1) correlated with the Injury Severity Score (ISS) and arterial base deficit (BD). Data are presented in quartiles, * *P *≤ 0.05 based on test for rank and trend.

There is experimental evidence that HMGB1 may be an early mediator of sterile inflammation induced by hypoxia and ischemia-reperfusion, although previous experimental and clinical studies have demonstrated its role as a late mediator of inflammation in sepsis [14]. However, whether plasma levels of HMGB1 are elevated early after severe trauma in humans is unknown. Our initial findings indicate that HMGB1 levels increase with increasing ISS (*P *< 0.0003 by rank and *P *< 0.0001 by trend) and base deficit (*P *= 0.0019 by rank and *P *< 0.0001 by trend). There is a strong positive correlation between HMGB1 and ISS r = 0.41, *P *< 0.0001), and a similar positive correlation between HMGB1 levels measured 30 minutes after severe trauma and base deficit (r = 0.35, *P *= 0.0003; Figures 1a and 1b). Interestingly there was a higher HMGB1 level in blunt trauma patients (11.70 ± 18.3) vs penetrating trauma victims (5.06 ± 8.6 *P *= 0.02).

### Plasma levels of HMBG1 and early systemic inflammatory response in trauma patients

**Figure 2:**
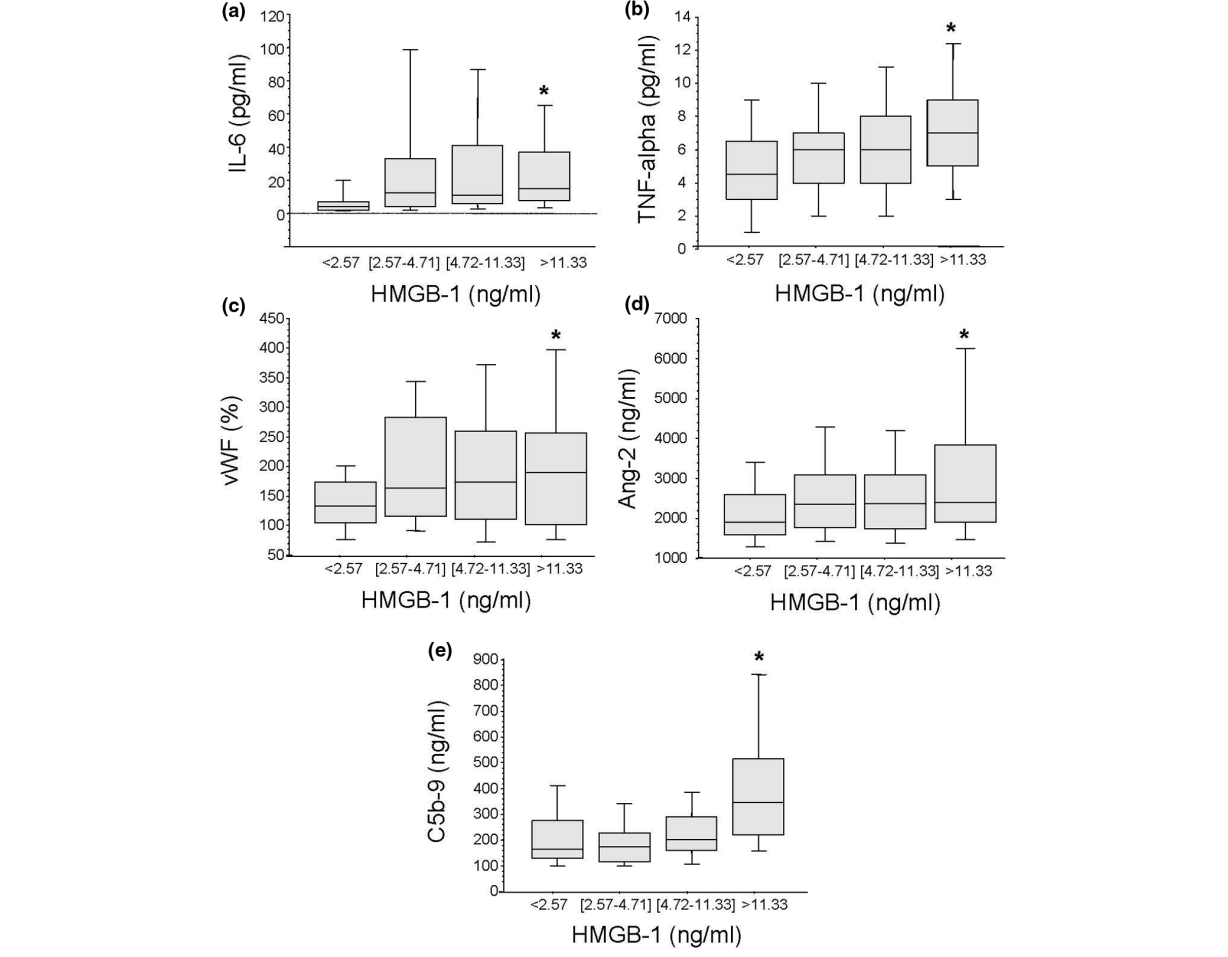
High plasma levels of HMGB1 are associated with the release of inflammatory mediators and markers of endothelial cell and complement activation in trauma patients. Blood samples were obtained from 168 consecutive major trauma patients immediately upon admission to the hospital. **(a to d) **Plasma levels of high mobility group box nuclear protein 1 (HMGB1) are associated with increased plasma levels of IL-6, TNF-α, Von Willebrand Factor (vWF) and angiopoietin-2 (Ang-2). **(e) **High plasma levels of HMGB1 are associated with increased complement activity as indicated by elevated soluble C5b-9 plasma levels that are generated during the late phase of complement activation. Data are presented in quartiles, *P ≤ 0.05 based on test for rank and trend.

Previous studies have shown that HMGB1 can cause the release of inflammatory mediators by several cell types including endothelial cells via the activation of TLR4 and RAGE. We thus determined the relationship between plasma levels of HMGB1 and inflammatory mediators early after trauma. Again, here HMGB1 levels increase with increasing levels of and early proinflammatory mediators such as IL-6 (*P *= 0.0001 by rank and *P *< 0.0001 by trend, Spearman correlation r = 0.36, *P *< 0.0001) and TNF-α (*P *= 0.03 by rank 0.004 by trend, Spearman correlation r = 0.25, *P *= 0.0013; Figures 2a and 2b). Ang-2 is stored in the same endothelial cell organelles as vWF (Weibel Palade bodies) and released in part by the same mechanism upon endothelial stimulation, such as hypoxia and ischemia-reperfusion associated with severe trauma [26]. We thus examined whether plasma levels of these markers of endothelial cell activation would correlate with those of HMGB1 early after trauma. We found HMGB1 increased with increasing plasma levels of vWF (*P *= 0.05 by both rank and trend, Spearman correlation r = 0.18, *P *= 0.02) and Ang-2 (*P *= 0.09 by rank but 0.01 by trend, Spearman correlation r = 0.23, *P *= 0.02; Figures 2c and 2d).

Recent experimental studies have indicated that alarmins, a family of early danger signal mediators to which HMGB1 belongs, and complement appear to be the early mediators of the sterile inflammatory response associated with hemorrhagic shock [14]. Furthermore, a recent experimental study has suggested that complement can activate the release of HMGB1 [27]. Finally, we have previously reported that there is an activation of complement within 45 minutes after severe trauma in humans [9]. We thus determined whether there was a correlation between activation of complement and plasma levels of HMGB1 within 45 minutes after trauma. The results indicate that trauma patients who had the higher plasma levels of HMGB1 had significantly higher plasma levels of C5b-9 (membrane attack complex) generated as the final common pathway of complement activation (*P *= 0.0001 by rank and trend, Spearman correlation r = 0.33, *P *= 0.0001; Figure 2e).

### Plasma levels of HMBG1 and early coagulation derangements in trauma patients

**Figure 3:**
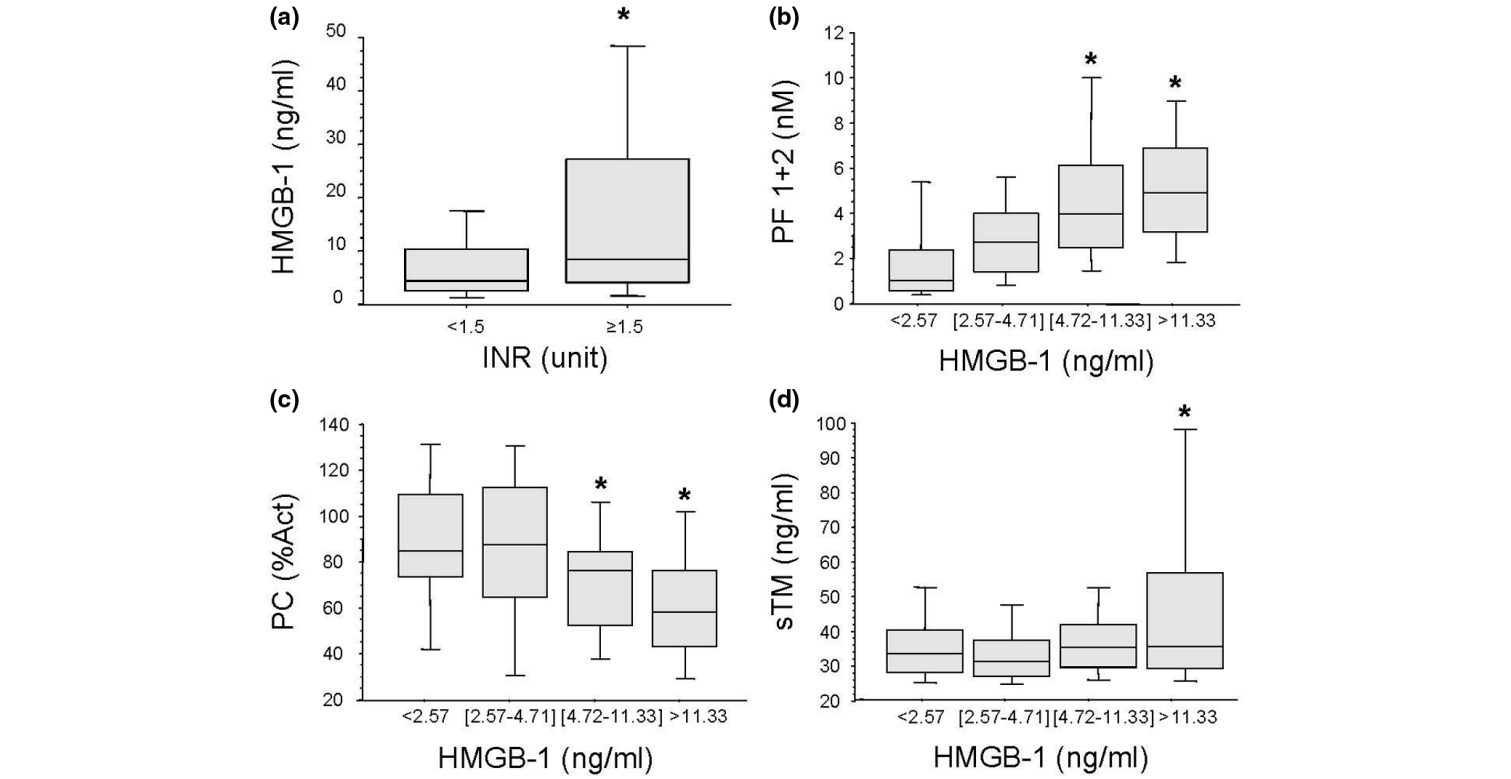
High plasma levels of HMGB1 are associated with coagulation abnormalities in trauma patients. Blood samples were obtained from 168 consecutive major trauma patients immediately upon admission to the hospital. **(a) **Trauma patients with coagulation abnormalities (international normalized ratio (INR) >1.5) had significantly higher levels of high mobility group box nuclear protein 1 (HMGB1). *P ≤ 0.05 from patients with INR <1.5. **(b to d) **High plasma levels of HMGB1 were associated with coagulation derangements early after trauma that are not due to coagulation factor deficiency as shown by the rise in the levels of soluble PF 1+2, a marker of thrombin generation and soluble thrombomodulin as well as a fall in protein C levels. Data are presented in quartiles, *P ≤ 0.05 based on test for rank and trend.

**Figure 4:**
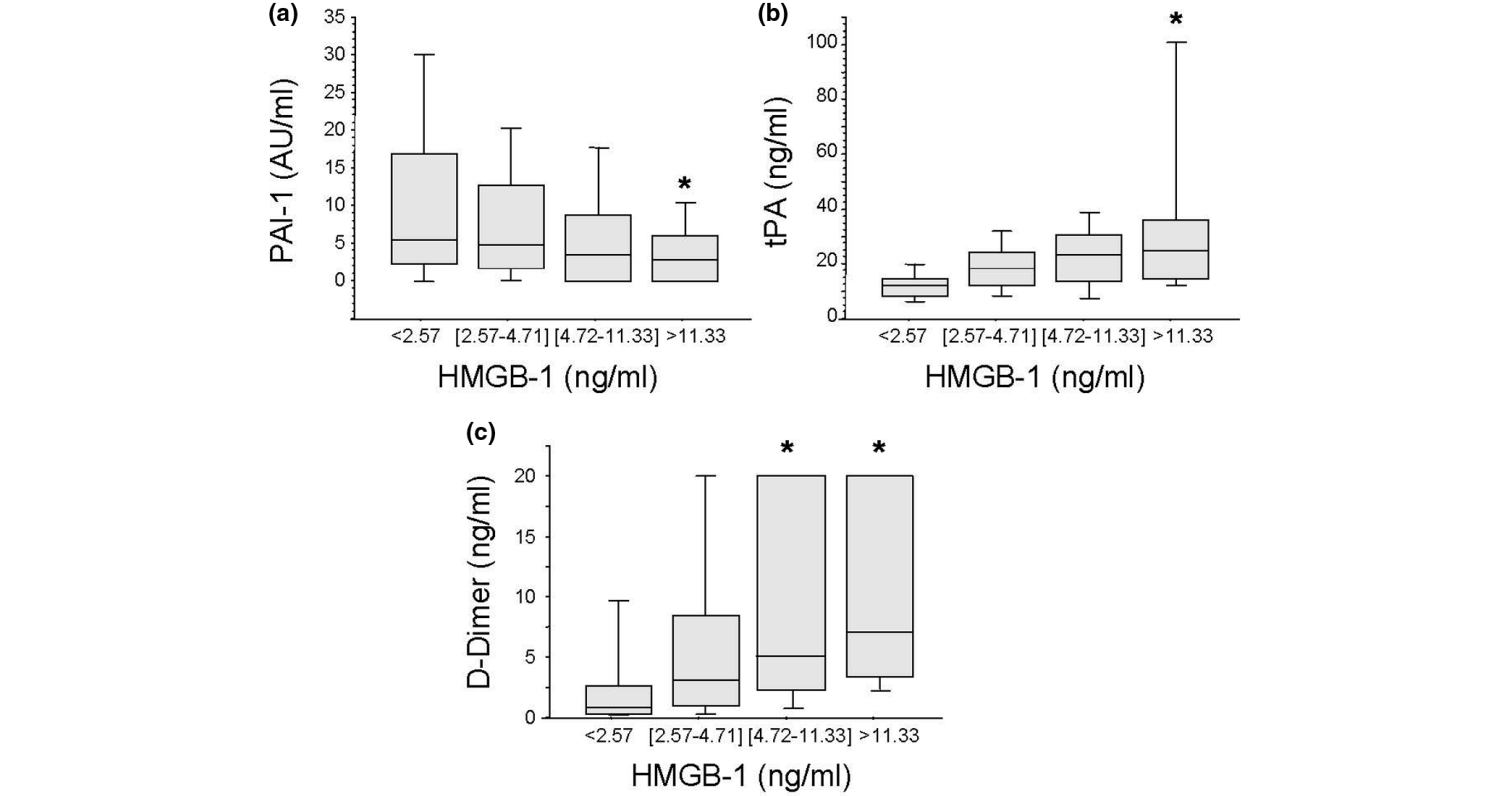
High plasma levels of HMGB1 are associated with increased fibrinolytic activity in trauma patients. Blood samples were obtained from 168 consecutive major trauma patients immediately upon admission to the hospital. **(a to c) **High plasma levels of high mobility group box nuclear protein 1 (HMGB1) are associated with increased fibrinolytic activity early after trauma, as shown by the plasma levels of plasminogen activator inhibitor-1 (PAI-1), tissue plasminogen activator (t-PA) and D-Dimers. Data are presented in quartiles, *P ≤ 0.05 based on test for rank and trend.

Coagulation abnormalities are common following major trauma and are directly related to worse clinical outcome [28]. We have recently shown that only patients who are severely injured and in shock are coagulopathic at the admission to the Emergency Department within 45 minutes after injury and that the development of this coagulopathy correlates with the activation of the protein C pathway rather than with the consumption of coagulation factors [20]. We next sought to identify whether the release of HMGB1 in our patients was related to coagulation abnormalities. Patients with clinically significant coagulation abnormalities (international nationalized ratio (INR) >1.5) had significantly higher plasma levels of HMGB1 (*P *= 0.01; Figure 3a). Furthermore, increasing plasma levels of HMGB1 were associated with a rise in INR (Spearman correlation r = 0.20, *P *= 0.008) the levels of soluble PF 1+2, a marker of thrombin generation (*P *= 0.001 by rank and *P *< 0.0001 by trend Spearman correlation r = 0.53 *P *≤ 0.0001), soluble thrombomodulin (*P *= 0.06 by rank and *P *= 0.02 by trend, Spearman correlation r = 0.24 *P *= 0.002) and a fall in protein C levels (*P *= 0.002 by rank and trend Spearman correlation -.39, *P *≤ 0.0001; Figures 3b to 3d). Finally, plasma levels of HMGB1 were negatively correlated with those of PAI-1 (*P *= 0.04 by rank and *P *= 0.03 by trend, Spearman correlation r = -.23, *P *= 0.004), and positively correlated with t-PA (*P *= 0.0001 by rank and trend, Spearman correlation r = 0.46, *P *≤ 0.0001 and D-Dimer levels (*P *= 0.001 by rank and trend, Spearman correlation r = 0.50, *P *≤ 0.0001; Figures 4a to 4c), suggesting an increased fibrinolytic activity in patients with elevated plasma levels of HMGB1.

### Plasma levels of HMGB1 and clinical outcome in trauma patients

**Figure 5:**
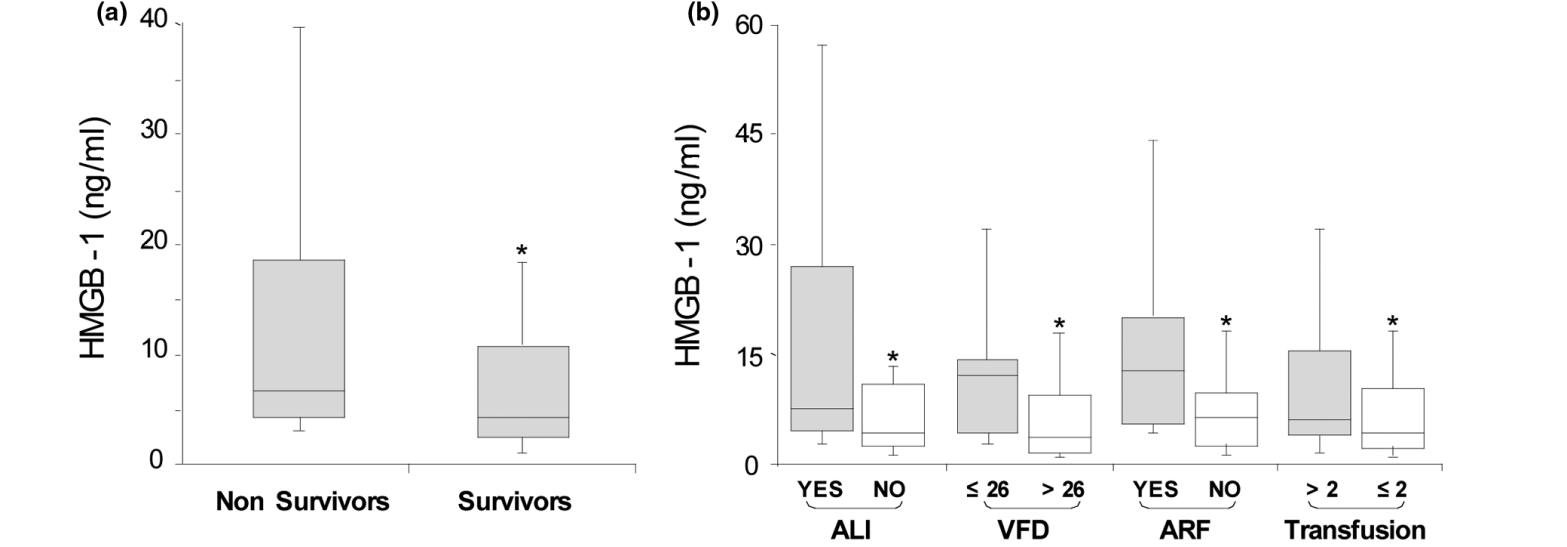
High plasma levels of HMGB1 are associated with increased mortality and end-organ injury in trauma patients. **(a) **Baseline plasma levels of high mobility group box nuclear protein 1 (HMGB1) after severe trauma were higher in non-survivors compared with survivors. Graphs depict median and interquartile range; *P *= 0.02 by Wilcoxon rank-sum). **(b) **Patients who developed acute lung injury (ALI) had significantly higher levels of plasma HMGB1 compared with those who did not develop ALI (median 7.49 vs 4.28 ng/ml, *P *= 0.02). Likewise, patients with fewer ventilator free days (VFDs) had higher plasma HMGB1 levels compared with those with more VFDs (*P *= 0.0004). Patients who developed acute renal failure (ARF) had significantly higher plasma HMGB1 levels compared with those who did not develop ARF (median 12.76 vs. 4.14 ng/ml, *P *= 0.0001). Patients who required more than two units of packed red cell transfusion also had higher plasma HMGB1 levels compared with those transfused with fewer units of blood (*P *= 0.03). Graphs depict median and interquartile range.

Finally, to determine the clinical significance of these findings, we examined whether HMGB1 release into the bloodstream within 30 minutes after injury was associated with worse clinical outcome. We found that there was a direct relation between mortality rate and plasma levels of HMGB1. A doubling of HMGB1 levels was associated with a 1.7 times likelihood of death (odds ratio 1.70; 95% confidence interval 1.12 to 2.60; *P *= 0.01; Figures 5a to 5b). Non-survivors (n = 26) had significantly higher than plasma levels of HMGB1 than survivors (n = 183; Figures 5a and 5b). Furthermore, patients who later developed organ injury, such as acute lung injury (n = 18) and acute renal failure (n = 23). had also significantly higher plasma levels of HMGB1 measured immediately after admission to the Emergency Department within 45 minutes after injury (Figure 5c).

## Discussion

The results of this study demonstrate for the first time that: (a) HMGB1, a known early mediator of sterile inflammation, is released in the plasma within 45 minutes after severe trauma in humans; (b) the release of HMGB1 in the plasma requires severe tissue injury and tissue hypoperfusion; and (c) HMGB1 is associated with posttraumatic coagulation abnormalities, activation of complement and severe systemic inflammatory response.

**Figure 6:**
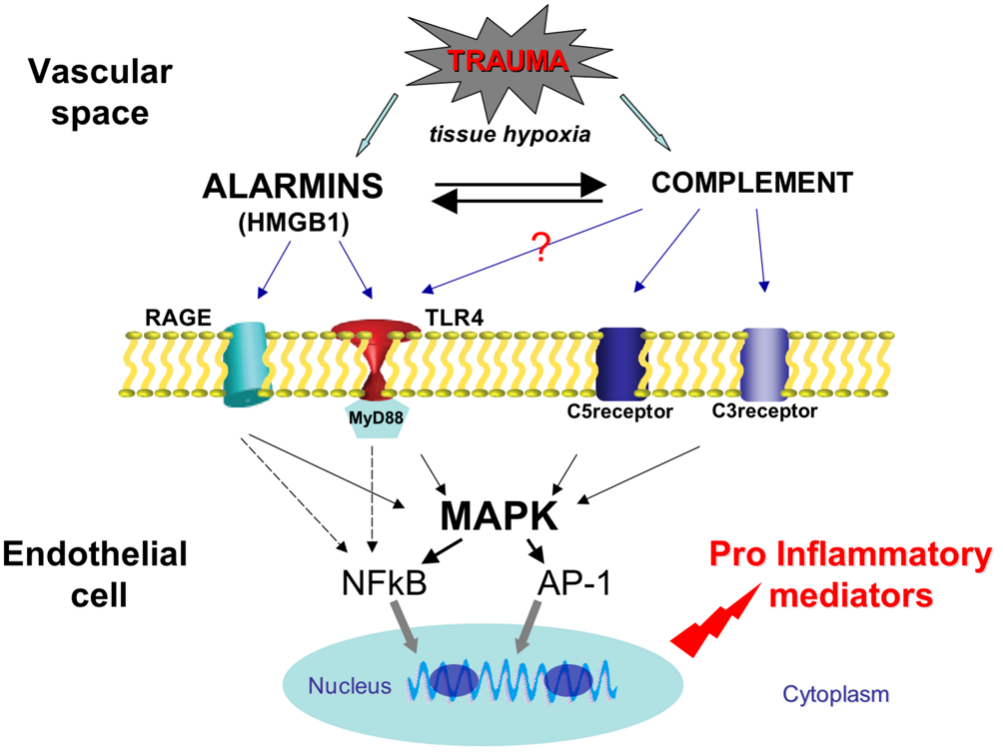
Schematic diagram: relation between the release of HMGB1, complement activation and induction of an inflammatory response in the vascular endothelium early after trauma. HMGB1 = high mobility group box nuclear protein 1; RAGE: receptor for the advanced glycation end products. MAPK: mitogen-activated protein kinases.

Severe trauma is associated with an early SIRS seen within 30 to 60 minutes after injury followed by a CARS observed 24 to 48 hours after injury, although the molecular mechanisms responsible for this altered host defense are not well understood [2-4]. Recent studies have provided new information on the molecular mechanisms that lead to the early inflammatory response. Complement and alarmins have been shown in experimental studies to play an important role as endogenous triggers of trauma-associated inflammation. Among the alarmins, HMGB1 appears to be one of the important mediators in triggering this posttraumatic sterile inflammation via receptors, such as TLR4 and RAGE [12-14,29] (Figure 6). However, whether HMGB1 is an early mediator of the early inflammatory response induced by severe trauma in humans is unknown. Only one previous study had described HMGB1 release in plasma in a small group of patients several hours after trauma [17]. We present here for the first time evidence that HMGB1 is released within 30 minutes after trauma in patients with severe injury and tissue hypoperfusion. There was no significant fluid resuscitation or other potentially confounding treatment prior to blood sampling and therefore our findings represent the direct effects of the injury and shock on the release of HMGB1 into the bloodstream.

Initial interest in HMGB1 as a biomarker of inflammation came from the work of Tracey and colleagues [17] who showed that HMGB1 was released in response to lipopolysaccharide (LPS) in mice. Significantly HMGB1 was released at a later time point (peak at 16 hours) as compared with the nearly immediate release of TNF-α and IL-1β after exposure to LPS. These findings were extended by the same research group who showed that HMGB1 is a factor of lethality in mice rendered septic by the induction of a polymicrobial bacterial peritonitis. Further studies reported that HMGB1 could induce the release of proinflammatory cytokines and induce an increase in permeability across intestinal cell monolayers [14]. The interest for this late release of HMGB1 after exposure to LPS was related to the fact that an anti-HMGB1 blocking antibody could rescue mice from lethality after cecal ligation and puncture as late as 24 hours after the beginning of sepsis [30,31]. In humans, plasma levels of HMGB1 have been shown to be elevated in ICU patients with sepsis and patients after major surgery (esophagectomy) [32]. Both Wang and colleagues and Sunden-Cullberg and colleagues reported a prolonged elevation of plasma levels of HMGB1 in septic patients [33,34]. Interestingly in these studies, there was no correlation between elevation in HMGB1 levels and severity of infection. In a more recent study, Gibot and colleagues reported that plasma levels of HMGB1 measured at day three after onset of severe sepsis discriminated survivors from non-survivors [35]. Taken together, these results indicate that HMGB1 is a late mediator of sepsis that has an important mechanistic role in that disease, because the inhibition of HMGB1 activity significantly ameliorates the survival in experimental animal models of septic shock.

In contrast to the data reported for sepsis, we found a significant difference in plasma levels of HMGB1 between survivors and non-survivors from severe trauma. This major difference in the plasma level profile of HMGB1 between septic and hemorrhagic shock may be explained by the fact that experimental studies have shown that HMGB1 is one of the alarmins, proteins that play a critical role in initiating the sterile inflammatory response after onset of ischemia-reperfusion injury [16]. The results of these experimental studies are supported by the correlation we found between plasma levels of HMGB1 and several inflammatory mediators, such as IL-6 and TNF-α, as well as markers of endothelial cell activation, such as Ang-2 and vWF antigen. Taken together, previous studies and our results indicate different kinetics for the release of HMGB1 during the two major causes of shock: sepsis and hemorrhage. HMGB1 appears to be an early mediator of the sterile inflammation induced by trauma-hemorrhage; in contrast, the kinetics of HMGB1 release due to sepsis may differ depending on the primary source of infection [34].

The second important result of our study is the relation between the plasma levels of HMGB1 and the activation of the protein C pathway that we have previously shown to be induced by tissue injury and hypoperfusion. This relation is particularly interesting in light of the recent discovery that HMGB1 binds *in vitro *to the lectin domain of TM. Abeyama and colleagues reported that TM could bind HMGB1 and serves thus as a sink for active HMGB1 in the plasma [36]. These results add to the concept that TM is an anti-inflammatory protein via its sequestration of thrombin, and its activation of protein C and Thrombin activated fibrinogen inhibitor (TAFI)[29]. Whether TM after binding HMGB1 would still maintain its ability to activate protein C is unclear, although protein C activation is dependent on the Gla domain of TM while HMGB1 is bound to its lectin domain. Ito and colleagues recently reported that administration of HMGB1 caused fibrin deposition and prolonged clotting times in healthy rats [19]. These investigators also showed that HMGB1-bound TM and thereby reduced the ability of thrombomodulin to activate protein C *in vitro*. In contrast to the results of these experimental studies, our current data show a simultaneous release of HMGB1 in the plasma and an activation of the protein C pathway by tissue injury and hypoperfusion suggesting that the release of HMGB1 in the plasma is not sufficient to inhibit the activation of the protein C pathway and the development of coagulopathy within 45 minutes after severe trauma-hemorrhage. However, these clinical results do not exclude that, in addition to the cytokine-like effect of HMGB1 via the TLR4 and RAGE receptors, extracellular HMGB1 could also attenuate the maladaptive activation of the protein C observed after severe trauma. Additional studies with a mouse model of trauma-hemorrhage that mimics the findings in trauma patients are needed to demonstrate this new function of extracellular HMGB1 after severe trauma and are currently being performed in our laboratory.

## Conclusions

In summary, the results of the present study indicate that HMGB1, a known early mediator of sterile inflammation, is released within 30 minutes after trauma in humans. Plasma levels of HMGB1 correlate with the severity of injury, tissue hypoperfusion, early posttraumatic coagulopathy and hyperfibrinolysis as well with a systemic inflammatory response and activation of complement. Patients who did not survive their injuries had significantly higher plasma levels of HMGB1 early after trauma than those who did. Future studies will be needed to determine whether the inhibition of HMGB1 early after trauma may significantly reduce the systemic inflammatory response associated with tissue injury and hypoperfusion.

## Key messages

• HMGB1 is elevated in plasma early after injury and shock in human patients.

• Plasma levels of HMGB1 correlate with injury severity and shock.

• Plasma levels of HMGB1 correlate with early post-traumatic coagulopathy and other markers of systemic inflammation.

• Early HMGB1 elevation is associated with increased morbidity and mortality in trauma patients.

## Abbreviations

Ang-2: angiopoietin-2; CARS: compensatory anti-inflammatory response syndrome; HMGB1: high mobility group box nuclear protein 1; INR: international nationalized ratio; ISS: injury severity score; LPS: lipopolysaccharide; PAI-1: plasminogen activator inhibitor-1; PAMPs: pathogen-associated molecular patterns; PF1+2: prothrombin fragments 1+2; RAGE: receptor for the advanced glycation end products; SIRS: systemic inflammatory response syndrome; TLR4: toll-like-receptor 4; TM: thrombomodulin; TNF-α: tumor necrosis factor alpha; tPA: tissue plasminogen activator; vWF: Von Willebrand Factor.

## Competing interests

The authors declare that they have no competing interests.

## Authors' contributions

MJC carried out the design, sample collection, measurement, analysis, and preparation of the manuscript. KB participated in sample collection, analysis and preparation of the manuscript. CC participated in data analysis and preparation of the manuscript. PR and BC participated in sample collection, measurement, analysis, and preparation of the manuscript. MC, SC and MH participated in analysis and preparation of the manuscript. JFP participated in the design, sample collection, measurement, analysis, and preparation of the manuscript. All authors read and approved the final manuscript.

## References

[B1] Mathers CD Loncar D Projections of global mortality and burden of disease from 2002 to 2030 PLoS medicine 2006 3 e442 10.1371/journal.pmed.0030442 17132052 PMC1664601

[B2] Moore FA Sauaia A Moore EE Haenel JB Burch JM Lezotte DC Postinjury multiple organ failure: a bimodal phenomenon The Journal of trauma 1996 40 501 510 discussion 510-502 10.1097/00005373-199604000-00001 8614027

[B3] Stahel PF Smith WR Moore EE Role of biological modifiers regulating the immune response after trauma Injury 2007 38 1409 1422 10.1016/j.injury.2007.09.023 18048034

[B4] Moore FA Moore EE Evolving concepts in the pathogenesis of postinjury multiple organ failure The Surgical clinics of North America 1995 75 257 277 7899997 10.1016/s0039-6109(16)46587-4

[B5] Cobb JP Buchman TG Karl IE Hotchkiss RS Molecular biology of multiple organ dysfunction syndrome: injury, adaptation, and apoptosis Surgical infections 2000 1 207 213 discussion 214-205 10.1089/109629600750018132 12594891

[B6] Bhole D Stahl GL Therapeutic potential of targeting the complement cascade in critical care medicine Critical care medicine 2003 31 S97 104 10.1097/00003246-200301001-00014 12544983

[B7] Arumugam TV Shiels IA Woodruff TM Granger DN Taylor SM The role of the complement system in ischemia-reperfusion injury Shock (Augusta, Ga) 2004 21 401 409 10.1097/00024382-200405000-00002 15087815

[B8] Guo RF Ward PA Role of C5a in inflammatory responses Annual review of immunology 2005 23 821 852 10.1146/annurev.immunol.23.021704.115835 15771587

[B9] Ganter MT Brohi K Cohen MJ Shaffer LA Walsh MC Stahl GL Pittet JF Role of the alternative pathway in the early complement activation following major trauma Shock 29 34 10.1097/shk.0b013e3180342439 17510601

[B10] Collard CD Vakeva A Morrissey MA Agah A Rollins SA Reenstra WR Buras JA Meri S Stahl GL Complement activation after oxidative stress: role of the lectin complement pathway The American journal of pathology 2000 156 1549 1556 10793066 10.1016/S0002-9440(10)65026-2 PMC1876913

[B11] Mollnes TE Fosse E The complement system in trauma-related and ischemic tissue damage: a brief review Shock 301 310 10.1097/00024382-199410000-00012 7757525

[B12] Bianchi ME DAMPs, PAMPs and alarmins: all we need to know about danger Journal of leukocyte biology 2007 81 1 5 10.1189/jlb.0306164 17032697

[B13] Klune JR Dhupar R Cardinal J Billiar TR Tsung A HMGB 1: Endogenous Danger Signaling Molecular medicine (Cambridge, Mass) 2008 14 476 484 10.2119/2008-00034.Klune PMC2323334 18431461

[B14] Fink MP Bench-to-bedside review: High-mobility group box 1 and critical illness Critical care 2007 11 229 10.1186/cc6088 17903310 PMC2556731

[B15] Sama AE D'Amore J Ward MF Chen G Wang H Bench to bedside: HMGB1-a novel proinflammatory cytokine and potential therapeutic target for septic patients in the emergency department Acad Emerg Med 2004 11 867 873 15289194 10.1197/j.aem.2004.03.011

[B16] Williams JH Ireland HE Sensing danger--Hsp72 and HMGB1 as candidate signals Journal of leukocyte biology 2008 83 489 492 10.1189/jlb.0607356 18156188

[B17] Yang R Harada T Mollen KP Prince JM Levy RM Englert JA Gallowitsch-Puerta M Yang L Yang H Tracey KJ Harbrecht BG Billiar TR Fink MP Anti-HMGB1 neutralizing antibody ameliorates gut barrier dysfunction and improves survival after hemorrhagic shock Molecular medicine (Cambridge, Mass) 2006 12 105 114 10.2119/2006-00010.Yang PMC1578769 16953558

[B18] Levy RM Mollen KP Prince JM Kaczorowski DJ Vallabhaneni R Liu S Tracey KJ Lotze MT Hackam DJ Fink MP Vodovotz Y Billiar TR Systemic inflammation and remote organ injury following trauma require HMGB1 American journal of physiology 2007 293 R1538 R1544 17652366 10.1152/ajpregu.00272.2007

[B19] Ito T Kawahara K Nakamura T Yamada S Nakamura T Abeyama K Hashiguchi T Maruyama I High-mobility group box 1 protein promotes development of microvascular thrombosis in rats J Thromb Haemost 2007 5 109 116 10.1111/j.1538-7836.2006.02255.x 17239166

[B20] Brohi K Cohen MJ Ganter MT Matthay MA Mackersie RC Pittet JF Acute traumatic coagulopathy: initiated by hypoperfusion: modulated through the protein C pathway? Annals of surgery 2007 245 812 818 10.1097/01.sla.0000256862.79374.31 17457176 PMC1877079

[B21] Baker SP O'Neill B Haddon W Jr Long WB The injury severity score: a method for describing patients with multiple injuries and evaluating emergency care The Journal of trauma 1974 14 187 196 4814394

[B22] Davis JW Parks SN Kaups KL Gladen HE O'Donnell-Nicol S Admission base deficit predicts transfusion requirements and risk of complications The Journal of trauma 1996 41 769 774 10.1097/00005373-199611000-00001 8913202

[B23] Rutherford EJ Morris JA Jr Reed GW Hall KS Base deficit stratifies mortality and determines therapy The Journal of trauma 1992 33 417 423 1404512 10.1097/00005373-199209000-00014

[B24] Bernard GR Artigas A Brigham KL Carlet J Falke K Hudson L Lamy M Legall JR Morris A Spragg R The American-European Consensus Conference on ARDS American journal of respiratory and critical care medicine 1994 149 818 824 7509706 10.1164/ajrccm.149.3.7509706

[B25] Bellomo R Ronco C Kellum JA Mehta RL Palevsky P Acute renal failure - definition, outcome measures, animal models, fluid therapy and information technology needs: the Second International Consensus Conference of the Acute Dialysis Quality Initiative (ADQI) Group Critical care (London, England) 2004 8 R204 R212 10.1186/cc2872 PMC522841 15312219

[B26] Ganter MT Cohen MJ Brohi K Chesebro BB Staudenmayer KL Rahn P Christiaans SC Bir ND Pittet JF Angiopoietin-2, marker and mediator of endothelial activation with prognostic significance early after trauma? Ann Surg 2008 247 320 326 10.1097/SLA.0b013e318162d616 18216540

[B27] Rittirsch D Flierl MA Nadeau BA Day DE Huber-Lang M Mackay CR Zetoune FS Gerard NP Cianflone K Kohl J Gerard C Sarma JV Ward PA Functional roles for C5a receptors in sepsis Nature medicine 2008 14 551 557 10.1038/nm1753 PMC2753858 18454156

[B28] Brohi K Singh J Heron M Coats T Acute traumatic coagulopathy The Journal of trauma 2003 54 1127 1130 10.1097/01.TA.0000069184.82147.06 12813333

[B29] Esmon C Do-all receptor takes on coagulation, inflammation Nature medicine 2005 11 475 477 10.1038/nm0505-475 15875050

[B30] Wang H Liao H Ochani M Justiniani M Lin X Yang L Al-Abed Y Wang H Metz C Miller EJ Tracey KJ Ulloa L Cholinergic agonists inhibit HMGB1 release and improve survival in experimental sepsis Nature medicine 2004 10 1216 1221 10.1038/nm1124 15502843

[B31] Ulloa L Ochani M Yang H Tanovic M Halperin D Yang R Czura CJ Fink MP Tracey KJ Ethyl pyruvate prevents lethality in mice with established lethal sepsis and systemic inflammation Proceedings of the National Academy of Sciences of the United States of America 2002 99 12351 12356 10.1073/pnas.192222999 12209006 PMC129448

[B32] Suda K Kitagawa Y Ozawa S Saikawa Y Ueda M Abraham E Kitajima M Ishizaka A Serum concentrations of high-mobility group box chromosomal protein 1 before and after exposure to the surgical stress of thoracic esophagectomy: a predictor of clinical course after surgery? Dis Esophagus 2006 19 5 9 10.1111/j.1442-2050.2006.00529.x 16364036

[B33] Wang H Bloom O Zhang M Vishnubhakat JM Ombrellino M Che J Frazier A Yang H Ivanova S Borovikova L Manogue KR Faist E Abraham E Andersson J Andersson U Molina PE Abumrad NN Sama A Tracey KJ HMG-1 as a late mediator of endotoxin lethality in mice Science 1999 285 248 251 10.1126/science.285.5425.248 10398600

[B34] Sunden-Cullberg J Norrby-Teglund A Rouhiainen A Rauvala H Herman G Tracey KJ Lee ML Andersson J Tokics L Treutiger CJ Persistent elevation of high mobility group box-1 protein (HMGB1) in patients with severe sepsis and septic shock Critical care medicine 2005 33 564 573 10.1097/01.CCM.0000155991.88802.4D 15753748

[B35] Gibot S Massin F Cravoisy A Barraud D Nace L Levy B Bollaert PE High-mobility group box 1 protein plasma concentrations during septic shock Intensive care medicine 2007 33 1347 1353 10.1007/s00134-007-0691-2 17525840

[B36] Abeyama K Stern DM Ito Y Kawahara K Yoshimoto Y Tanaka M Uchimura T Ida N Yamazaki Y Yamada S Yamamoto Y Yamamoto H Iino S Taniguchi N Maruyama I The N-terminal domain of thrombomodulin sequesters high-mobility group-B1 protein, a novel antiinflammatory mechanism The Journal of clinical investigation 2005 115 1267 1274 15841214 10.1172/JCI22782 PMC1077171

